# Inheritance vs. infectivity as a mechanism of malady and death of Frederic Chopin

**DOI:** 10.1007/s13353-021-00651-2

**Published:** 2021-08-04

**Authors:** Michał Witt, Tadeusz Dobosz

**Affiliations:** 1 Institute of Human Genetics, Polish Academy of Sciences, Strzeszynska 32, 60-479, Poznan, Poland; 2 Institute of Forensic Medicine, Wrocław Medical University, Wrocław, Poland

**Keywords:** Chopin, Cystic fibrosis, Heart, Pericarditis, Tuberculosis

## Abstract

Based on a macroscopic analysis of the heart of Frederic Chopin performed in 2014, it can be stated with high probability that the composer suffered from a long lasting tuberculosis as a primary disease, which was the cause of progressive deterioration of his physical condition and numerous symptoms mainly from the respiratory tract. Tuberculous pericarditis rapidly progressing within a rather short period of time, a relatively rare complication of diffuse tuberculosis, might have been an immediate cause of death. This would aptly coincide with a startling opinion that in an autopsy picture the composer’s heart had been more affected by the disease than the lungs.

Of all the biographical accounts of Frederic Chopin, born in 1810, with many uncertain or even anecdotal elements, one is evident: the composer was of poor health and chronically ill with a disease whose main symptoms came from the airways. The disease was manifested by a persistent cough and periodic shortness of breath, expectoration of thick, and sticky secretions and hemoptysis, which appeared in a later stage of the illness. Family history tells us that the composer’s younger sister Emilia died at the age of 14, also from a symptomatically very similar but rapid and more severe lung disease.

Frederic Chopin died in Paris in 1849, in the presence of his older sister Ludwika Jędrzejewicz. As for the cause of his death, we can only relate to questionably legitimate hypotheses. The Chopin’s autopsy protocol, which was carried out by the famous physician and anatomist Jean Cruveilhier, was burned in a fire in the Paris police archives in 1871. We can only rely on the vague accounts of Chopin’s former student Jane Stirling. It is known that the autopsy review presented a surprising statement on pathological changes more pronounced in the heart than in the composer’s lungs. In accordance with Chopin’s will, his heart was removed from the corpse. From accounts, we know of the composer’s patriotic-emotional desire to have his heart returned to Poland after his death; the medical version, devoid of such idealization, not excluding it though, points to possible taphophobia (psychopathology involving fear of being buried alive). Frederic Chopin’s funeral was very solemn; his body was laid to rest at Père-Lachaise cemetery in Paris.

A year after Chopin’s death, his sister Ludwika transported the heart, preserved, and secured in a special jar, to Warsaw, supposedly smuggling it across the Russian border under the folds of her dress. It was transferred to the Holy Cross Church, the Chopins’ home parish; after long-term storage in the church catacombs, in 1880, it was placed in the crypt in the upper church. It remained there until the Warsaw Uprising of 1944, when it was carried out of the partially ruined church by a Wehrmacht officer, the divisional chaplain, and then handed over with military honors by SS General Erich von dem Bach-Zelewski into the hands of the Bishop of Warsaw, Rev. Antoni Szlagowski. The whole ceremony was commemorated for propaganda purposes by a German newsreel.

In 1945, the heart was finally returned to the crypt in the nave pillar of the Holy Cross Church—the ceremony of bringing Chopin’s heart back became one of the first events during the reconstruction of Warsaw, destroyed by the war; the symbolic value of this ceremony was used by the authorities of those times to promote the new government and political system. The heart of Frederic Chopin rests to this day in this location.

## Chopin’s course of disease

The first symptoms of the disease appeared in Chopin already in his teens; it was diagnosed as an unspecified inflammation of the airways, “catarrhal affection”; of note, swelling of the neck lymph glands (mycobacterial cervical lymphadenitis?) occurred in a 16-year-old Frederic. Since then, the composer’s illness progressed with periodical exacerbations. From 1838, symptoms started to intensify, limiting pianist’s and composer’s life and artistic activity, and in the last 2 years of his life, they became extremely severe, what finally led to his death at the age of 39. Since then, the final diagnosis of Chopin’s disease remained speculative, being an unsolved mystery in the pathography of the most outstanding and famous Polish composer.

Given the multitude and severity of symptoms of Chopin’s disease, the most probable diagnosis, the pulmonary tuberculosis, has been repeatedly invoked. However, many authors have also put forward other concepts and hypotheses, such as mild-course cystic fibrosis (Majka et al. 2003; Persson et al. 2005), alpha-1-antitrypsin deficiency causing emphysema (Kuzemko 1994), mitral stenosis (O’Shea 1990), and several others, more or less medically substantiated (idiopathic bronchiectases, primary ciliary dyskinesia, hypogammaglobulinemia, allergic bronchopulmonary aspergillosis, tricuspid valve incompetence, Churge-Strauss syndrome, pulmonary hemosiderosis, pulmonary arteriovenous malformation) (Neumayr 1997; Lagerberg 2011). All of the aforementioned diseases are characterized by similar non-specific clinical symptoms, such as chronic cough, dyspnea, or general weakness.

Such symptoms as chronic, progressive course (from early adolescence), suffocating cough with expectoration of large amounts of sticky secretion and further hemoptysis (George Sand vividly wrote “Chopin spits out washbasins of blood”), significant emaciation and poor tolerance of physical strain, recurrent inflammation of the lower and upper airways (including sinuses and larynx), the need to follow a fat-free diet (dietary errors caused for him severe diarrhea), heatstroke (on Mallorca), symptoms of arthropathy (of pulmonary origin?), no offspring despite a long relationship with Sand (infertility?), a similarly symptomatic lung disease in his sister Emilia (the occurrence of the disease in only one generation is characteristic of the autosomal recessive inheritance, as is for cystic fibrosis). To these theoretical *ex post* reflections, it should be added, however, that the well-known cast of Chopin’s left hand, made after the composer’s death in 1849 by the sculptor August Clesinger, son-in-law of George Sand, does not indicate the presence of “clubbed fingers”, typical for full-blown pulmonary cystic fibrosis (disease symptom consisting of deformation of the nails and thickening of the distal phalanges, which take on a characteristic shape; the cause is hypoxia of the peripheral parts of the body).

## Inspection of the crypt containing Chopin’s heart

The only earlier description of Frederic Chopin’s preserved heart, drawn up in 1951 by Bronisław Edward Sydow, the Chopin musicographer, author of the first Chopin’s bibliography and initiator of Chopin’s Festival in Duszniki-Zdrój, for obvious reasons has a limited significance as a source of medical information. It is based on observations made during the ceremony of placing the urn with Chopin’s heart in the crypt at the Holy Cross Church in May 1945, when the jar was inspected for the last time. Since then, for 69 years, the heart was stored without any monitoring, being a routine safety requirement in museum collections of anatomical preparations. Hence, an inspection of the object to check its preservation status was fully justified. The prerequisite was to exclude any invasive activity to the object itself and to focus exclusively on macroscopic analysis and photographic documentation of the heart. The adopted non-destructive method of proceeding a priori excluded the possibility of carrying out a genetic analysis of Chopin’s heart. Such a procedure ensured the necessary safety of the object itself and protection from potentially fatal effects of physical interference.

The inspection was attended by the Archbishop of the Warsaw Metropoly, Card. Kazimierz Nycz, the Minister of Culture and National Heritage, Bogdan Zdrojewski, the Directors of the National Frederic Chopin Institute (NIFC), Dr. Artur Szklener, and Dr. Wojciech Marchwica, Rector of the Holy Cross Church Parish in Warsaw Rev. Zygmunt Berdychowski and two medical professionals, Prof. Tadeusz Dobosz from the Department of Forensic Genetics, Medical University, Wroclaw, and Prof. Michal Witt from the Institute of Human Genetics, Polish Academy of Sciences, Poznan. Accompanying persons from the church and the NIFC, a technical team and a photographer were also present.

The inspection itself lasted about an hour and was carried out in the late evening, after the church had finally closed, with full confidentiality. A detailed photographic documentation was performed; however, the examiners were unable to use any additional diagnostic equipment (e.g., CT or MRI scanner). The result of the macroscopic analysis of Frederic Chopin’s heart was exclusively described previously by us (Witt et al. 2018a).

In the crypt, beneath the decorative headboard, was a light brown wooden box, oak in appearance, with a hinged lid with a security device, covered at the top with a thick paper. The box had the initials F.C. engraved on the lid. Inside was another box, black in color (mahogany in appearance), lacquered, with decorative metal embossing along all walls. Attached to the lid was a heart-shaped silver plate with engraved inscription: *F. Fryderyk Chopin. Urodzony w Polsce d. 1. Marca 1810 r. Umarł w Paryżu w d. 17 października 1849 r*. (F. Frederic Chopin. Born in Poland on 1 March 1810. Died in Paris on 17 October 1849). Between the outer brown case and the inner black case were lead plates upholstered in purple velvet (most likely originally black). In a specially formed cylindrical opening, there was a crystal jar of about 1.25–1.5 L capacity, containing a heart in a conservation fluid. The jar was tightly closed with a ground glass plug, protected by an externally applied seal of vaseline. Some of it seeped into the inside of the jar. Judging by its traces on the inner wall of the vessel, the liquid level has dropped by at most 0.5 cm since the moment of closing, which proves the excellent tightness of the ground plug used.

The heart inside was immersed in an amber-colored conservation fluid, covering it with an excess of about 2–3 cm—it is known from historical sources that it may have been a wine distillate (in diluted consumable form known as brandy), which matches with the color of the liquid. In those days, it was a relatively easily accessible solution of ethanol with a concentration of about 60%. Since the time of the French Revolution, this solution was used for tissue preservation—a fortunate coincidence that such a concentration is still considered optimal for this purpose.

## Morphology of Chopin’s heart

After more than 170 years, the state of preservation of Frederic Chopin’s heart is still excellent. It fills about ¾ of the jar’s volume; the overall appearance clearly indicates a significant hypertrophy of the organ. The heart is incomplete; several anatomical structures are missing: the left atrium and most of the right atrium together with blood vessels, except for the so-called right auricula, the trunks of the main artery, and the pulmonary artery. The color of the myocardium is typical of fixed muscle.

The general heart appearance corresponds to the morphological features of *cor pulmonale*, with significant enlargement of the right ventricle; the left ventricle is enlarged to a lesser extent. In the anterior wall of the left ventricle, a long dissection incision is visible, which allowed an insight into its lumen and an assessment of its interior and the condition of the mitral valve; the incision was stitched with several surgical sutures. Previously, little attention was paid to a postmortem examination of the right heart, which by today’s criteria presents a deviation from the proper autopsy technique. Right ventricular hypertension, a consequence of pulmonary hypertension, has led to a right ventricular myocardial hypertrophy and dilatation, most likely accompanied by a right atrial hypertension, hypertrophy of its walls, and/or dilatation. Such a hypertrophy of the myocardium (*hypertrophia cordis*) results in extensive changes in the myocardium: altered myocyte thickness and length, decreased capillary density, increase in connective tissue with no systolic function, increased metabolic requirements, which altogether lead to increased vulnerability to decompensation. Such extensive cardiomegaly is usually accompanied by symptoms of myocardial failure. The appearance of Chopin’s heart suggests its chronic, mainly right ventricular, failure.

The lack of vessels of *corona cordis* (aorta and pulmonary trunk) and the absence of atria may be explained by the postmortem technique used in France and the satellite countries, differing from the “German-Austrian”; the previous involves cutting off the heart from the rest of thoracic organs at the level of *corona cordis* and examining it separately. In the “German” technique, the whole set of organs (heart, lungs, esophagus) is removed from the chest and then each of them is examined separately (Dérobert 1974; Piédelièvre and Fournier 1963). Because Chopin’s heart was enlarged and flaccid at the time of the autopsy, the incision was overly low, leading to damage of both atria. Hemorrhagic effusions, probably as a result of the presence of a subendocardial bloody fluid typical of tuberculous pericarditis, were clearly visible, mainly on a wall of the left ventricle.

Neither calcifications nor adhesions were found on the epi- and pericardium. The entire epicardium was covered with a white massive fibrillary coating; loose precipitates of the coating are apparent as flocs freely floating in the conservation fluid. Numerous small villi on the cardiac surface showed features of fibrinous pericarditis. The most prominent lesions found were three small nodules, between several millimeters up to 1 cm in diameter, of white-glass appearance: two of them on the upper part of the right ventricular wall, one located closer to the apex of the heart, most likely of tuberculous origin.

## Macroscopic data analysis

The nodules present on the surface of Chopin’s heart look like myocardial tuberculoma described previously (Rosenbaum and Linn 1948). With the current level of TB therapy, such advanced organ lesions do not occur in well-developed countries. Similar lesions may also occur in the course of sarcoidosis, but this cause would not be justified in context of Chopin’s medical history.

The formation of such nodules and tapestry-like white coating by *post mortem* processes was ruled out (Witt et al. 2018b). These lesions, on closer inspection, differ significantly from focal mold colonies and deposits of inorganic crystals. Mold colonies are characterized by well-defined sharp edges; crystals appear only locally as separate foci; they also differ entirely from local lipid deposits. Such lesions occur mainly in old objects with significantly decreased levels of conservation fluid which was not the case for Chopin’s heart. White epicardial coating was widespread fairly evenly over the surface and was clearly visible regardless of the angle of observation, being a proof of an inflammatory process affecting the entire organ. Such morphology makes up the picture of fibrinous pericarditis, described in some sources as *frosted heart* or *bread and butter appearance*. The epicardial morphology argues for a short duration of pericarditis with a rapid progression. In contrast, prolonged tuberculous pericarditis is characterized by pericardial calcification with adhesions and is usually constrictive in nature (*pericarditis constrictiva*).

The morphologic data obtained in our study (Witt et al. 2018a,b), described exclusively by us, and the data from the composer’s natural history indicate with high probability that in the last period of his life Frederic Chopin suffered from severe fibrinous pericarditis, as evidenced by fibrinous deposits on its surface, foci of hyalinization, focal hemorrhagic effusions on the left ventricular anterior wall, dilatation of mainly the right ventricle and probably the right atrium, indicative of the right ventricular chronic heart failure. We can state with high probability that for Chopin, his active tuberculosis was complicated by pericarditis which thus should be considered tuberculous pericarditis (*pericarditis tuberculosa*). It is one of the most severe organ complications of disseminated tuberculosis, characterized by extremely poor prognosis (Mayosi 2009). Fibrinous deposits on the epicardium, together with hemorrhagic effusions that sometimes appear, are specific especially for a subacute or early chronic phase of the disease (Mayosi 2009; Kotharii and Roy 2009; Tavora and Burke 2017).

## Tuberculosis in Chopin’s time

In the nineteenth century, tuberculosis was endemic in Northern Europe. In the group of children/youth under 18 years of age, as much as 90% were infected with tuberculosis. Mortality rate due to tuberculosis in the general population was colossal, amounting to 25–30% of all deaths in those days. This rate did not change in a significant positive way until the early twentieth century, due to civilizational improvements in living conditions (Lawn and Zumla 2011). In Chopin’s time, tuberculosis was romantically idealized as an elusive inspiration for painters, writers, or musicians under the name *spes phthysica* (“tubercular hope”), as a trigger for the euphoric creative energy that was attributed to consumptives. This is a primary example of the relationship, widely described and discussed in psychology, between suffering caused by illness and a particular upsurge of the human spirit in the form of unbridled, creative artistic inspiration (Abbott 1982).

Cardiovascular tuberculosis is a rare complication of the tuberculosis and accounts for only 1–3% of extra-pulmonary tuberculosis cases. It is currently the leading cause of pericarditis worldwide and occurs most frequently in developing countries, mainly as a complication of HIV infection. Pericardial involvement in the course of tuberculosis is most often acquired from mediastinal lymph nodes, more rarely, by blood or through the continuity of foci located in adjacent organs. The majority of patients present with exudative form, more rarely with exudative-restrictive or restrictive form. The first symptoms are usually fever, weight loss, night sweats, almost always cough and dyspnea, and chest pain. In the acute stage of tuberculous pericarditis, there is an accumulation of exudate in the pericardial sac. As the fluid builds up, dyspnea develops; the presence of fluid in the pericardial sac impairs effectively cardiac function. Symptoms of circulatory failure become evident: deterioration of exercise tolerance, liver enlargement, swelling of the lower extremities, presence of pleural fluid which additionally aggravates dyspnea. The development of constrictive pericarditis results in increasing circulatory failure (Tavora and Burke 2017). The above catalog of lesions and symptoms was in its entirety documented in the descriptions of the natural history of Frederic Chopin’s disease and fully corresponds with the results of macroscopic analysis of the heart conducted by us in 2014.

## Cause of death

On the basis of our macroscopic analysis, it may be stated with a high degree of certainty that the primary disease from which Frederic Chopin suffered was prolonged disseminated pulmonary tuberculosis. This became the cause of the progressive deterioration of the composer’s general physical condition and of many different symptoms, mainly of the airways. Tuberculous pericarditis, rapidly progressing in the last period of his life, could have been the direct cause of the composer’s death by leading to circulatory and respiratory failure.

This almost conclusively solves the problem of Frederic Chopin’s illness and cause of death. The fact that his younger sister Emilia died with similar clinical symptoms shows the contingency of household spread of the disease rather than being a proof of the familial occurrence of a genetic disorder like cystic fibrosis, primary ciliary dyskinesia, or alpha-1-antitrypsin deficiency. It can be assumed that Emilia suffered from miliary tuberculosis, a prognostically unfavorable manifestation of pulmonary tuberculosis caused by hematogenous dissemination of tuberculous infection over lungs and other organs.

## What else can be done?

In conclusion, although chronic caverneous pulmonary tuberculosis, accompanied by laryngeal and intestinal involvement, is the most probable diagnosis of Chopin, and tuberculous pericarditis and rapidly progressing cardiopulmonary insufficiency the direct cause of death, it is only genetic analysis that can provide definitive and irrefutable proof of other hypotheses. In particular, this applies to the exclusion/confirmation of rare genetic diseases: cystic fibrosis, alpha-1-antitrypsin deficiency or primary ciliary dyskinesia, as Chopin’s primary disease. As already mentioned, in 2014 such an analysis, based on the preserved heart tissue of the composer, could not be made. Considering the benefit of this priceless object, regarded by Chopin’s compatriots as a national relic, this must not be allowed to happen now or ever in the future. Any possible opening of the jar will cause a fundamental change in the physical, chemical, and microbiological environment inside, which would pose a dramatic threat to the state of preservation of the so far perfectly conserved tissue. The next inspection of the state of conservation of Chopin’s heart, in accordance with the routine applied in anatomical collections, is recommended around 2065.

From the genetic point of view, however, there is still a way to carry out such analyses—a source of material may be remains of the parents Justyna (née Krzyżanowska) and Mikołaj Chopin, as well as his sister Emilia at Stare Powązki cemetery in Warsaw. Non-destructive extraction of a miniscule amount of genetic material from bones or teeth can be performed during the already necessary renovation of gravestones. It would be the most appropriate finale, completing the picture obtained on the basis of our research to date, being of utmost historical, cultural, and medical significance.

## Data Availability

N/A.

## Code availability

N/A
